# Lymphopenia With Clinical and Laboratory Features of Combined Immune Deficiency in an 11-Year-Old Female With *FANCD2* Variants and Fanconi Anemia

**DOI:** 10.3389/fped.2018.00390

**Published:** 2019-01-18

**Authors:** Roman Deniskin, Ghadir S. Sasa, Sarada L. Nandiwada, Nicholas L. Rider

**Affiliations:** ^1^Department of Pediatrics (Pediatrician Scientist Training and Development Program), Houston, TX, United States; ^2^Section of Hematology and Oncology, Baylor College of Medicine and Texas Children's Hospital, Houston, TX, United States; ^3^Clinical and Diagnostic Immunology, Baylor College of Medicine and Texas Children's Hospital, Houston, TX, United States; ^4^Section of Allergy, Immunology, and Rheumatology, Baylor College of Medicine and Texas Children's Hospital, Houston, TX, United States

**Keywords:** immune deficiency, Fanconi anemia, FA-D2, FANCD2, recombination

## Abstract

Fanconi anemia (FA) is an inherited bone marrow failure and cancer predisposition disorder due to mutations in DNA repair pathways proteins (FANC). The dysfunctional proteins are unable to repair DNA breaks and cause genomic instability. Mutations in many of the 19 *FANC* genes are well characterized biochemically and clinically. Little is known about the *FANCD2* gene which acts downstream of the FA-core proteins. Here we report a 11-year-old female previously diagnosed with FA and bone marrow failure. Gene sequencing demonstrated deletion of exons 2-18 and a pathologic missense mutation (c. 2444G>A, p. Arg815Gln) in *FANCD2* (Chr3). Her medical history is significant for an episode of pneumococcal sepsis despite adequate vaccination. Repeated blood samples and immunophenotyping demonstrated severe lymphopenia. There were markedly low CD4^+^ T-cell counts with a low CD4:CD8 ratio. Changes in the composition of the B-cell population included significantly diminished absolute total B-cells, and decreased mature cells. There was no immunogenic response to vaccination against *S. pneumoniae*. The NK-cell count was unaffected and demonstrated normal spontaneous and stimulated cytotoxic response. Bone marrow analysis demonstrated hypocellularity without dysplasia. The clinical and laboratory features are suggestive of combined immune deficiency. FANCD2 may be involved in the transition of immature B and T cells to mature cells, a process that requires substantial DNA recombination not observed in NK cells. Additional genetic and biochemical evaluation is needed to further characterize the novel genetic and clinical findings.

## Introduction

Fanconi anemia (FA) is a rare (1:100,000–250,000 births) genetically and phenotypically heterogenous group of disorders caused by mutations in *FANC* genes. FANC proteins repair DNA damage incurred during normal cell replication, from radiation exposure, or due to chemotherapeutic agents (e.g., cisplatin, mitomycin C). Autosomal recessive or X-linked mutations induce genomic instability and cause congenital defects including microcephaly, cardiac, and genitourinary malformations, radial-ray defects, and intellectual disability. To date, 19 *FANC* genes have been identified in the FANC-mediated DNA-repair pathway (1). A comprehensive review of the repair mechanism is available elsewhere (2, 3). Briefly, a multi-subunit complex is assembled (FANC -A, -B, -C, -E, -F, -G, -L, and -M) at DNA breaks/lesions where replication has stalled (4). The FA-core complex activates FANCL which ubiquitinates the FANCI-FANCD2 complex. Mono-ubiquitination of FANCD2 is required for recruitment of repair proteins for nucleolytic incisions (5). *FANCA* mutations make up 60–65% of all FA patients worldwide. An additional 20% are due to defects in *FANCC* and *FANCG*. Comparatively, mutations in *FANCD2* (OMIM #613984) are rare, making up ~3% of all FA cases.

FA is a well-characterized cause of inherited bone marrow failure syndrome (BMFS). Furthermore, mutations in *FANC* genes or hypermethylation of the promoter sequences of the *FANC* genes predispose individuals to various hematological and non-hematological malignancies [e.g., head and neck squamous cell carcinoma (HNSCC), acute myelogenous leukemia (AML)] (6). In fact, one third of FA diagnosis is made at the time of concurrent AML diagnosis (7, 8).

Mutations in FA genes also affect immune cell development and function. In 1977, Pederson et al. reported a child with FA who had a primary immune deficiency affecting T-cell function (9). Since then, there has been steadily growing evidence of FA genes being involved in cell-mediated and humoral immunity. Immune dysregulation in Fanconi patients may be similar to other syndromes where the hallmark defect is chromosomal instability (e.g., Bloom, Nijmegen Breakage, Dyskeratosis Congenita). Few studies have examined clinical and immune characteristics associated with pediatric FA (10–12). To date, no studies have evaluated gene-specific changes in FA in relation to distinct immune deficiency phenotypes. In this report, we evaluated the immunological status of a patient with a novel *FANCD2* genotype. We identified defects in B and T cell lymphocytes, while sparing NK cell number and function. These results suggest a role for *FANCD2* in B and T cell development and contributes to combined immune deficiency.

## Materials and Methods

### Patient Demographic and Clinical Information

We conducted a comprehensive retrospective review of electronic medical records from outside facilities and those available at Texas Children's Hospital (Houston).

### Clinical Laboratory Assays

Phenotyping of peripheral blood mononuclear cells (PBMC) from freshly drawn anticoagulated (EDTA) whole blood samples were analyzed by a flow cytometric method. NK cell cytotoxicity was evaluated using ^51^Chromium release assay (CRA) modified from Nagel et al. (13). Briefly, NK cells (E = effector) were isolated from PBMCs and incubated for 4 h with K562 target cells (T = target; lack MHC Class I; monocyte lineage) prelabeled with ^51^Cr. Incubation was done in the presence or absence of IL-2. ^51^Cr released from lysed cells was measured using a gamma counter. NK cell killing frequency was calculated from the E:T ratio required to achieve 10% cytotoxicity.

### Genetic Testing

Blood samples were collected from our pediatric patient (9yo at the time), her biological mother, and her biological father with consent and under BCM Institutional Review Board Protocols. Samples were submitted to Invitae™ for Sanger sequencing of 17 genes from the Invitae Fanconi Anemia Panel (*BRCA2, BRIP1, ERCC4, FANCA, FANCB, FANCC, FANCD2, FANCE, FANCF, FANCG, FANCI, FANCL, FANCM, PALB2, RAD51C, SLX4, XRCC2*). The assay achieved >99% sensitivity and specificity for single nucleotide variants (SNPs) and insertions/deletions <15 bp.

### Consent for Publication

All the co-authors were aware of the journal's policy regarding publication of case material and have fulfilled it to the best of their knowledge. Parental consent was obtained for the publication of the manuscript and accompanying medical information.

## Results

**Figure 1:**
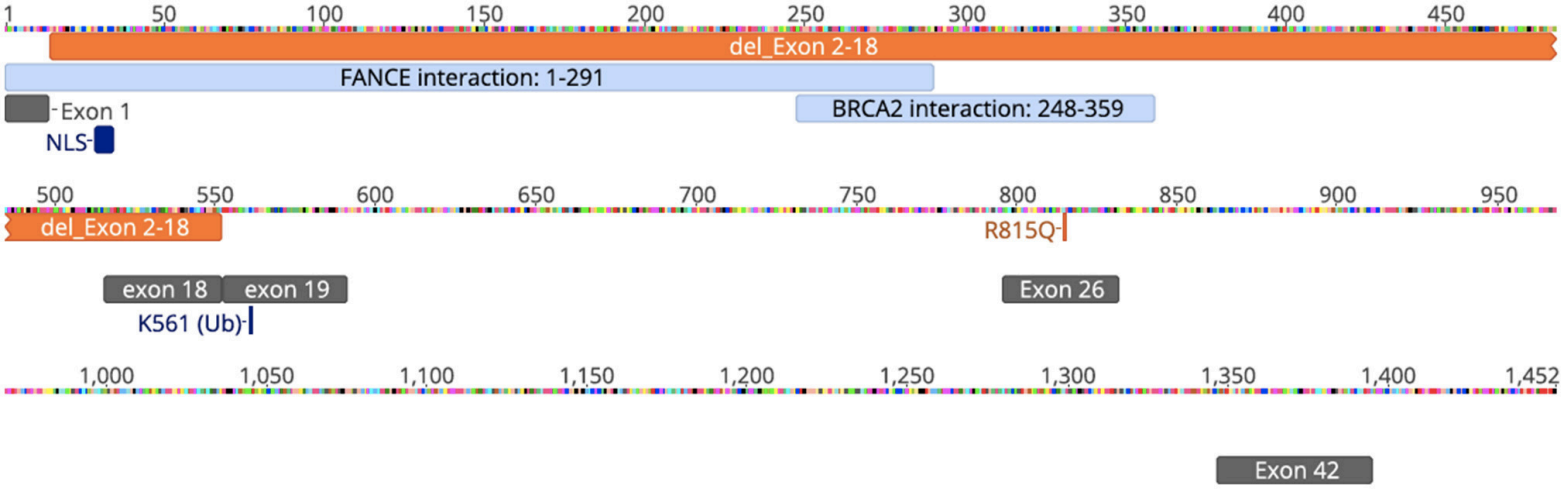
Schematic of FANCD2 protein sequence with pathologic alterations. FANCD2 is a 1,452 amino acid protein composed of 44 exons. Orange annotations represent our patient's genetic mutations (deletion of exons 2-18 and R815Q missense mutation; schematic does not reflect that these changes are on different alleles). Cyan annotations show regions of the protein important for protein-protein interaction with the FA-core complex (FANCE) and repair enzymes (BRCA2). Gray annotations represent various exons for helpful for orientation. NLS, or nuclear localization sequence, is in exon 2. Lysine-561 (K561) is located in exon 19 and is the sole site for ubiquitination and activation of FANCD2. Schematic was generated using Geneious® software v11.1.5. The annotations of interacting domains are based on data available from UniProtKB (ID: Q9BXW9) and BioGrid3.4 (ID:108474).

**Figure 2:**
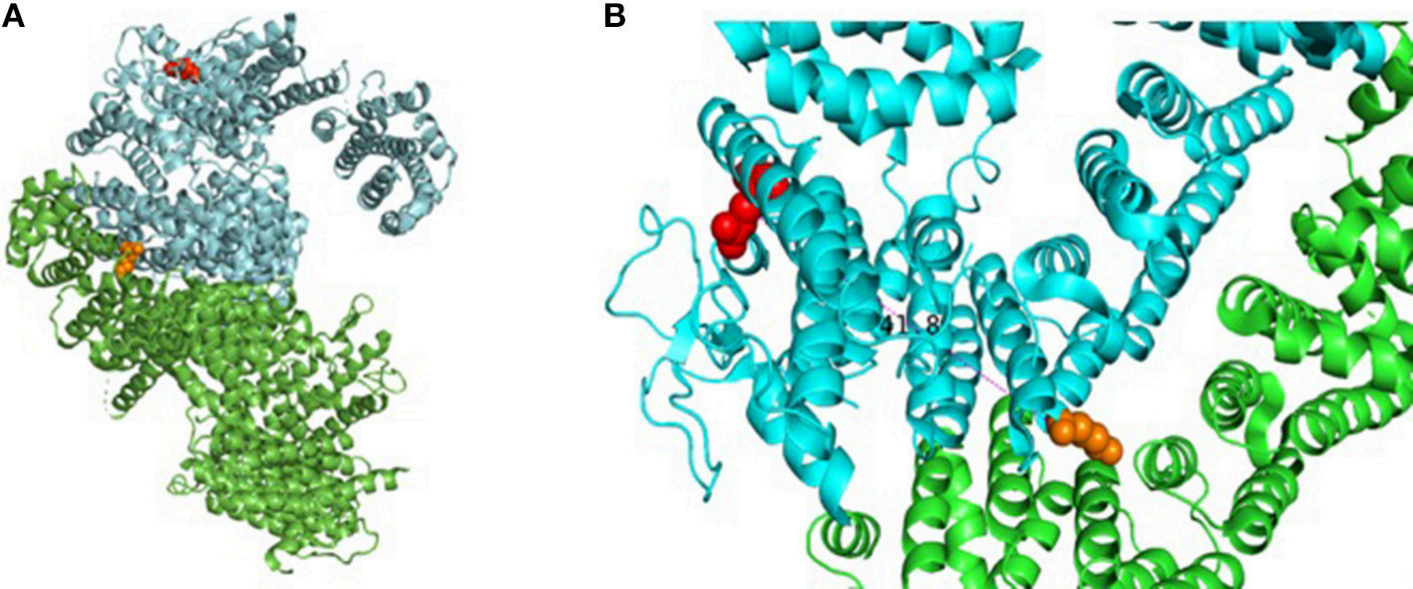
Assembly of FANCD2 and details of pathogenic missense mutation. **(A)** Cartoon drawing showing assembly of FANCD2 (cyan) in complex with FANCI (green) based on available crystal structure data (PDB 3S4W). Red amino acid residue represents location of R812Q on the protein surface; orange residue shows location of K561, which is essential for ubiquitination. **(B)** Closer look at R812Q on the protein surface relative to loop domains. Molecular models were generated using PyMol Molecular Graphics System (v2.2.0).

Our patient is an 11-year-old, Hispanic female who was born at 38 weeks gestational age via C-section. She presented with numerous congenital anomalies including mesomelia and radial ray defects (Table S1, Figures S1, S2). The postnatal course was complicated by total anomalous pulmonary venous return (TAPVR) cardiac defect that was repaired in the first week of life. DNA breakage studies of blood lymphocytes with diepoxybutane (DEB) were diagnostic of Fanconi anemia. There was no family history of blood disorders. Over a period of 11 years she has been evaluated in multiple institutions by various subspecialties, including hematology, cardiology, endocrinology, and otolaryngology. She had a history of multiple febrile illnesses the last few years, without any proven bacteremia or atypical infections, and HIV was excluded. Last year she developed *S. pneumoniae* sepsis requiring hospitalizataion despite receiving vaccination and had one episode of human metapneumovirus (hMPV) infection. The patient and her biological parents underwent genetic analysis to evaluate for mutations in FA pathway genes. Our patient has a c.2444G->A mutation in exon 26 of *FANCD2* leading to the missense mutation, R815Q (Figure 1). This is a known pathologic mutant previously reported in 17 FANCD2 patients, mostly of Hispanic ethnicity (76%), with an allele frequency of 0.01455% (ExAC variant 3:10108951 G/A; http://exac.broadinstitute.org/). Additionally, our patient has deletion of exons 2-18 of FANCD2 on the second allele, which is the largest *FANCD2* deletion reported thus far. Her biological father is Hispanic and an asymptomatic heterozygous carrier of the R815Q mutation. Her biological mother is also asymptomatic and carries a heterozygous deletion of exon 2–18. The R815Q mutation is located in exon 26, residing ~42 Å away from K561 and the FANCI-interacting region (Figure 2).

Comprehensive immunophenotyping was done when she was 10 years old while she was being evaluated for stem-cell transplantation. Immunophenotyping revealed severe B- and T- cell population abnormalities (Table 1). CD4^+^ counts were ~15% of the lower range of normal contributing to a moderately depressed CD4:CD8 ratio (0.41). Both naïve and memory CD4 helper T-cells were decreased. There was no effect on the number of NK cells in the peripheral blood. Absolute CD19^+^ B cell numbers were significantly reduced while the immunoglobulin defects were qualitative in our patient. She had been vaccinated before but had low vaccine titers, even after a PCV13 vaccine challenge. Mitogen proliferation assays showed poor response to phytohemaggluttinin (PHA), normal response to pokeweed Mitogen (PWM), and normal NK cell function (Table 1, Figure S3). A bone marrow aspiration and biopsy showed hypocellular marrow (overall 10% cellularity with trilineage hematopoiesis; <1% CD19^+^CD20^+^ B cells, 9% CD3+ T cells, <1% blasts, and absent plasma cells) and no significant dysplasia (data not shown). The results were consistent with mild-to-moderate bone marrow failure. Chromosomal analysis of the bone marrow revealed no clonal abnormalities. Fluorescence *in situ* hybridization (FISH) evaluation showed no cytogenetic changes suggestive of myelodysplastic syndrome (data not shown).

**Table 1:** Clinical basic and immune-specific phenotyping.

**Cell subset (units)**	**Normal range**	**Patient**
**CBC**		
WBC (x10^3^/μL)	5–14.5	2
ANC (x10^3^/μL)	1.5–8	1.18
Monocytes (%)	0–5	4.1
RBC (x10^3^/μL)	4–5.2	2.22
MCV (fL)	76–90	106.8
Hgb (g/dL)	11.5–15.5	7.9
Retic (%)	0.6–1.9	4.2
Plt (x10^3^/μL)	150–450	53
**Humoral Immunity (mg/dL)**		
IgA	66–295	106
IgG	641–1,353	862
IgM	40–80	34.7
**Post-vaccination Ig (IU/mL)**		
Tetanus	>1	0.3
Diptheria	>1	0.1
Varicella	>1	0.135
PCV13^*^	>1.5	<1.5^*^
**B-lymphocytes (x10**^**3**^**/μL)**		
CD3^−^ CD19^+^ (Total B cells)	270–860^†^	14
CD3^−^ CD19^+^ CD20^−^ (CD20^+^ B cells)	262–860^†^	14
CD3^−^ CD19^+^ CD38^L^CD21^L^ Imm	0–19	3
CD3^−^ CD19^+^ CD38^++^ IgM^++^ Tran	0–21	3
CD3^−^ CD19^+^ CD27^−^ IgM^+^ IgD^+^ Naive	120–430^‡^	11
CD3^−^ CD19^+^ CD27^+^ IgM^+^ IgD^+^ NSM	20–70^‡^	1
CD3^−^ CD19^+^ CD27^+^ IgM^+^ IgD^−^ SM	6–16^‡^	0
CD3^−^ CD19^+^ CD27^+^ IgM^−^ IgD^−^ CSM	30–110^‡^	0
CD3^−^ CD19^+^ CD21^+^ Mature	238–860^‡^	9
CD3^−^ CD19^+^ CD38^++^ IgM^−^ PB	0–6	1
CD3^−^ CD19^+^ CD27^+^ Mem	50–200^‡^	1
**T- and NK- lymphocytes (x10**^**3**^**/μL)**		
CD3^+^ T cells	991–2,997^†^	468
CD3^+^ HLD-DR^+^ T activated	0–250	216
CD3^+^CD4^+^ Th cells	635–1,620^†^	98
CD3^+^CD8^+^ Tc cells	293–1221^†^	241
CD4:CD8	0.7–2.6^†^	0.41
CD3^+^ CD4^+^ CD45RA^+^ Th naïve	320–1,000^‡^	12
CD3^+^ CD4^+^ CD45RO^+^ Th memory	230–630^‡^	72
CD3^+^ CD8^+^ CD56^+^ NKT8	30–200	90
CD3^−^ CD56^+^ CD16^+^ NK	0–200^†^	90
**Mitogen proliferation assay (counts x10**^**3**^**)**		
PHA	127–458^†^	93
PWM	53–222^†^	66
**NK cell cytotoxicity (killing frequency)**		
Unstimulated	>0.011^†^	0.06
IL-2 stimulated	>0.05^†^	0.13

*Imm, immature; Mem, memory; NSM, non-switched memory; PB, plasmablast; SM, switched memory; Tc, T-cytotoxic; Th, T-helper; Tran, Transitional*.

* *< 1.5 for all serotypes assessed (1,3,4,5,6B,7F,8,9N,9V,12F,14,18C,19F,23F)*.

† *Age-matched, TCH laboratory normal values*.

‡ *Age-matched, published normal values*.

## Discussion

This report presents the case of an 11-year old girl with novel compound heterozygous mutation/deletion of *FANCD2* and a new diagnosis of probable combined immune deficiency based on ESID criteria (Table S2). It is the first comprehensive immunophenotyping study of a patient with a *FANCD2* mutation. Among FA patients, the relative prevalence of *FANCD2* mutations is ~3%, with >1,400 known variants in the gene. *FANCD2* is a 1,452 amino acid protein made up of 44 exons. Most documented *FANCD2* gene changes produce splice variants or synonymous mutations. The patient had two unique genetic findings: a deletion of exons 2-18 on allele-1 and a R812Q missense mutation on allele-2. Exon 2 contains a positively charged, evolutionary-conserved bifunctional nuclear-localization signal (NLS) and DNA-binding region (SKKTK) (14). Mutations or deletion of this amino acid cluster causes defective nuclear foci formation. Deletion of exon 17 confers protein loss of function (15). Hence, her large exonic deletion is predicted to form a dysfunctional protein based on previous studies. Based on crystallography data, R812Q is located on the protein surface and is ~42 Å from K561, the sole ubiquitination site at the interface of FANCD2 and FANCI (Figure 2). The mutation converts a basic residue to an acidic residue on the protein surface. The native guanidinium group of arginine allows for ionic interaction in three directions while the glutamine offers interaction in two directions. The exact contribution of this mutation is unclear at this time, especially since the only ionic interactions that occur happen with the carbonyl backbone of the adjacent random loops. It is unknown if the change in the charge of the amino acid allosterically affects the DNA-binding domain which is 42 Å away.

The physical phenotype observed from our patient's R812Q mutation is similar to a small cohort of patients previously reported by Kalb et al. (16). In their study, four patients were compound heterozygous for the R812Q mutation. Two patients, siblings from a consanguineous relationship, were homozygous for the mutation and had physical manifestations similar to our patient (e.g., radial ray defects, skin pigmentation, absent ear canal). At this time, it is unclear if the R812Q mutation affects protein expression and folding, ubiquitination, interaction with FANCI, nuclear foci formation, or DNA repair. We are currently evaluating FANCD2 protein expression from our patient's PBMCs and hope to further characterize the mutation in an orthologous expression system. Overall, in the absence of functional protein due to the exonic deletion, we hypothesize that the R812Q mutated copy produced the severe congenital defects and lymphopenia affecting only the B and T cell populations. Further evaluation of FANCD2's role in DNA repair and other pathways may explain this unique immune-phenotype.

During lymphocyte proliferation (normal development or in response to antigen activation) the cells undergo DNA replication and recombination. In the process, the cells spontaneously produce DNA breaks and lesions. DNA breaks are repaired by either homologous recombination (HR) or non-homologous end joining (NHEJ) (15). Homologous recombination (HR) uses high-fidelity repair mechanisms and is dependent on FANC proteins. In contrast, NHEJ uses recombination activating gene (RAG) proteins and does not use the sister template DNA for high-fidelity repair. NHEJ is required for antigen receptor gene assembly in B- and T- lymphocytes via V(D)J recombination. NK cells contain germline-encoded antigen receptors and do not undergo V(D)J recombination (17, 18). Hence, mutations in the NHEJ pathways result in B- and T- cell dysregulations and produce a combined immune deficiency phenotype. Interestingly, defects in RAG1/2 and NHEJ proteins affect NK cell function but do not affect NK cell maturation (19, 20). Dobbs et al. showed that these NK cells had increased degranulation and higher perforin content (21). In this report, we demonstrated that our pediatric patient had severely reduced CD4^+^ T cells and B cells while the NK cell population was quantitatively and qualitatively normal. These findings are consistent with defects in NHEJ pathway and potentially explain why the patient was generally protected against viral illnesses. Our clinical findings expand the role of FANC proteins beyond HR and are in agreement with recent scientific evidence.

Recent findings provide convincing evidence that FANC proteins are involved in NHEJ. Pace et al. demonstrated that FANCC interacts with the NHEJ factor Ku70/80 to initiate NHEJ (22). Renaud et al. demonstrated that loss-of-function defects in FA core complex and FANCD2-deficient cells favors DNA repair using NHEJ (23). Inversely, FANCD2 modifies double-strand breaks to prevent Ku70/80 binding and partially inhibits NHEJ (19, 24). At this time, the balance of the dichotomous roles of the FA-core complex and FANCD2 in DNA repair is not clear and needs further investigation.

Animal models have validated the role of FANC proteins in immunity. Genetic knockout of FANCC in a murine model causes impaired differentiation of B-cells with concomitant decrease in antibody secretion (25). In human studies, compound homozygous and heterozygous mutations in FANCA and FANCE can lead to common variable immunodeficiency (CVID) (26). Roxo et al. demonstrated that FA patients (unknown genotypes) had poor response to pneumococcal vaccine (27). Defects in adaptive humoral immunity can be due to absolute B-cell lymphopenia and/or defective B-cell differentiation. The latter can be affected by both improper V(D)J recombination as well as a poor T-cell-dependent B-cell differentiation. Using a murine model of FANCA^−/−^, Nguyen et al. demonstrated that disruption of FANCA adversely affects primary diversification of immunoglobulins by hindering the IgM^−^ to IgM+ transition of B-cells (28). Our patient had clinical evidence of B-cell lymphopenia and defective maturation of peripheral B-lymphocytes (normal transitional but significantly decreased naive cells, near absent memory cells, and absent switched memory cells). These cells have already undergone Rag-dependent V(D)J recombination but seem to have stalled at the level of somatic hypermutation (SHM) and immunoglobulin class-switching recombination (CSR). Since FANCD2 is highly expressed in the germinal centers of lymphatic tissue where CSR and HSM occurs, it is possible that FANCD2 plays a role in CSR (28–30).

Previous reports of immune phenotyping of FA patients provided strong evidence for defective T, B, and NK cell compartments, the latter two being the most frequently encountered (10, 11, 31). Interestingly, Myers et al. had several patients with normal NK cell number but blunted cytotoxic function that correlated with diminished cell perforin and granzyme production (11). These studies, however, did not genetically characterize the different FA patients. It is possible that our patient's normal NK cell counts and function may ultimately become dysregulated and is in the spectrum of pan-lymphocyte defects seen in other FA patients. Additionally, her immunologic data reflects her most recent evaluation and her physiological state may change over time. Alternatively, her unique mutation may produce a novel phenotype where only the B and T cell compartments are affected. Recent clinical studies have showen that B and NK cell defects in FA patients occur earlier in life before T-cell defects (32). The absence of any NK cell dysregulation in our patient, even in the setting of bone marrow failure, suggests that this may indeed represent a novel phenotype.

More evidence is emerging about non-canonical roles of FANCD2. Buck et al. showed that T cell fate is controlled by mitochondrial metabolic programming (33). FANCD2 activates mitochondrial α-F_1_-ATP synthase (ATP5A) and controls cellular ATP levels (34). FANCD2 also interacts with elements of the mitochondrion-nucleoid complex to regulate mitochondrial homeostasis and biosynthesis (32). These findings may offer new insights into a new metabolic mechanism by which FANCD2 dysregulates immune cells.

Our clinical case study has several important limitations. First, it is challenging to interpret lymphopenia in the context of bone-marrow failure syndrome, especially as it relates to cellular maturation/development. The median age of BMFS in FA patients is typically at 7 years of age and unfortunately our evaluation was done at the age of ten. Second, our NK cell data does not reflect the phenotype and function of distinct NK cell subsets which may be altered (35). Third, our patient had a cardiothoracic surgical repair in infancy and most likely had a thymectomy (not documented in medical records). This is corroborated by the absence of a thymus on a recent chest CT scan. While FA alone can cause T cell dysregulation, we do not know to what extent an athymic state contributed to her overall immune deficiency. Finally, at this time we do not know the precise nature by which the R812Q mutation affects SHM and CSR—more work remains to be done to characterize this defect at the molecular level.

At this time, our patient is doing well and getting treatment with pentamidine and IVIG. Aside from the single episode of sepsis requiring hospitalization, she has not had any other serious infections while being evaluated for a bone marrow transplant. This case emphasizes the need for early identification of immune deficiency in patients with FA because these clinical findings may precede or exist independently of bone marrow failure and can alter clinical management strategies. This case also highlights the importance in recognizing that different FA genotypes (mutations in FA-core proteins vs. FANCD2 or other downstream proteins) may produce varying lymphocyte immune-phenotypes that contribute to different types of immune deficiencies. Therefore, molecular characterization of FA defects may lead to better understanding of the disease process.

## Ethics Statement

This study was carried out in accordance with the recommendations of the BCM Institutional Review Board Protocols with verbal assent from the pediatric patient (minor) and written informed consent from a legal guardian in accordance with the Declaration of Helsinki. The protocol was approved by the Baylor College of Medicine IRB.

## Author Contributions

RD, SN, GS, and NR obtained and analyzed the clinical data. RD and NR wrote the manuscript. GS and SN aided with manuscript edits. Immunophenotyping and cytotoxicity assays were conducted at the Clinical and Diagnostic Immunology Laboratory under the direction of SN.

### Conflict of Interest Statement

The authors declare that the research was conducted in the absence of any commercial or financial relationships that could be construed as a potential conflict of interest.
