# Supplementary material for: Lymphopenia With Clinical and Laboratory Features of Combined Immune Deficiency in an 11-Year-Old Female With FANCD2 Variants and Fanconi Anemia
Source: Front Pediatr. 2019 Jan 18;6:390. doi: 10.3389/fped.2018.00390 (PMC6346677; doi:10.3389/fped.2018.00390)

**SUPPLEMENTAL FIGURES AND TABLES**

**Lymphopenia with Clinical and Laboratory Features of Combined Immune Deficiency in an 11-year-old Female with *FANCD2* Variants and Fanconi Anemia.**

Running Title: Pediatric FANCD2 with Immune Deficiency

Roman Deniskin^1^, Ghadir S. Sasa^2^, Sarada L. Nandiwada^3,4^, and Nicholas L. Rider^4*^

^1^ Department of Pediatrics (Pediatrician Scientist Training and Development Program),

^2^ Section of Hematology and Oncology,

^3^ Clinical and Diagnostic Immunology

^4^ Section of Allergy, Immunology, and Rheumatology

Baylor College of Medicine and Texas Children’s Hospital, Houston (TX), USA

*Correspondence:

Dr. Nicholas L. Rider

email: nlrider@bcm.edu

mailing address:

Texas Children’s Hospital, Feigin Center, Suite 300

1102 Bates Avenue, Houston Texas, 77030

Section of Immunology, Allergy, and Rheumatology

Department of Pediatrics, Baylor College of Medicine

Key words: immune deficiency, Fanconi anemia, FA-D2, FANCD2, recombination

| **Table S1: Clinical and genetic manifestations (by system).** |
| --- |
| **CV**: *total anomalous pulmonary venous return (TAPVR)*  **MSK**: *microcephaly, L aural atresia, dysplasia of modiolous, mesomelia, radial ray dysplasia*  **Neuro**: *intellectual disability, optic neuropathy, Chiari malformation, central white matter loss*  **ID**: *S. pneumococcus sepsis, hMPV infection*  **Endo**: *short stature, ectopic neurohypophysis, growth hormone deficiency*  **Gene**: *Allele 1 – del_exon2-18; Allele 2 – R812Q* |

| **Table S2: Clinical Criteria for a Probable Diagnosis of Combined Immunodeficiency** |
| --- |
| Criteria adopted from ESID Registry Clinical Criteria Guidelines:  <https://esid.org/Working-Parties/Registry-Working-Party/Diagnosis-criteria>  ONE OR MORE OF:   - Severe infection requiring hospitalization ($\geq1$) - Evidence of immune dysregulation   - Autoimmunity, severe atopic dermatitis, inflammatory bowel disease, granuloma, lymphoproliferative disease - Malignancy - Affected family member   **AND**  TWO OR MORE OF:   - T-cell criteria   - Reduced, age-appropriate CD3 or CD4 or CD8 T-cell counts   - Reduced naïve CD4 and/or CD8 T-cell counts   - Elevated Gamma/Delta (γδ) T-cell counts   - Reduced mitogen-induced proliferation or TCR stimulation   **AND**  HIV excluded  **AND**  Exclusion of other clinical diagnosis associated with CID   - For list of CID-associated syndromes, see <https://www.uptodate.com/contents/combined-immunodeficiencies> |

**Figure S1. Longitudinal growth charts.**  Weight (A) and height (B) over time for our patient compared to CDC projected curves (P3 = 3^rd^ , P50 = 50^th^ , and P97 = 97^th^ percentiles for girls between ages 2- 20 yr). Most recent weight and stature were 16.8 kg (<0.01%, Z = -6.2) and 115cm (<0.01%, Z = -4.35), respectively*.*

**Figure S2. Non-contrast brain MRI.** *Left* Sagittal section demonstrating central white matter loss, thinning of corpus callosum, and Chiari I malformation. *Right* Coronal section demonstration absent left ear canal.

**
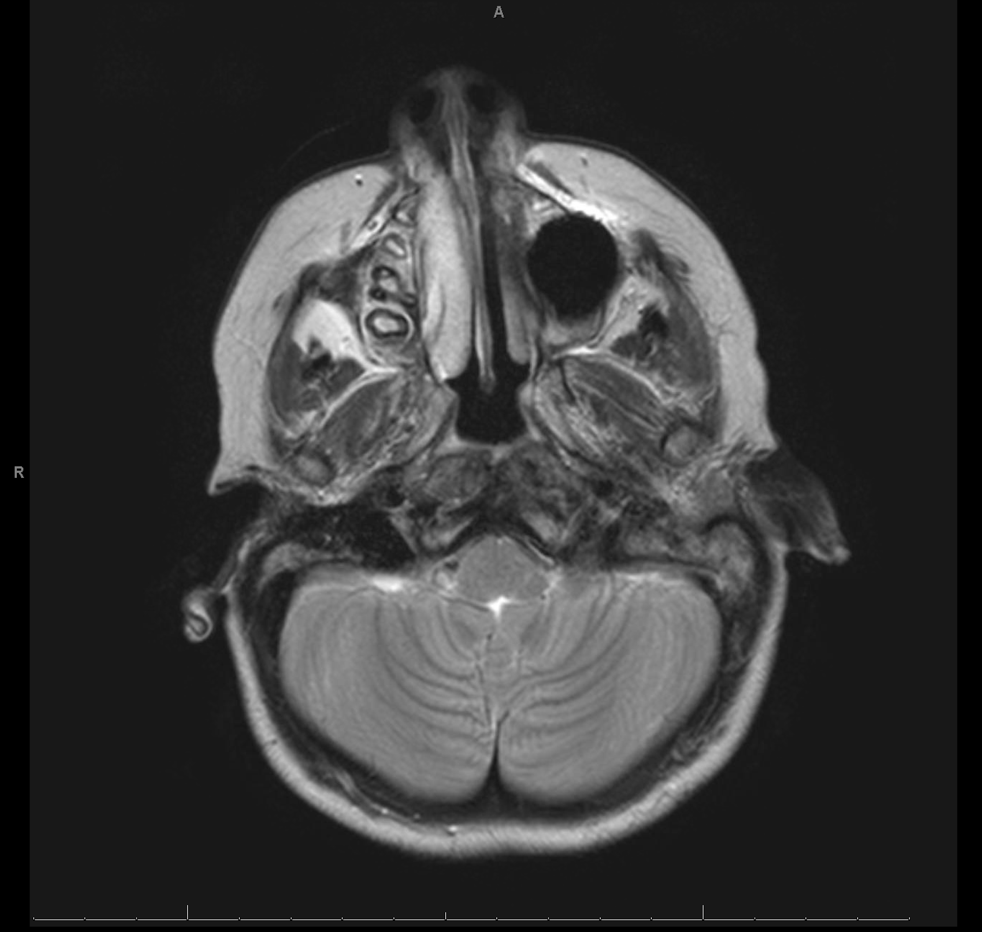

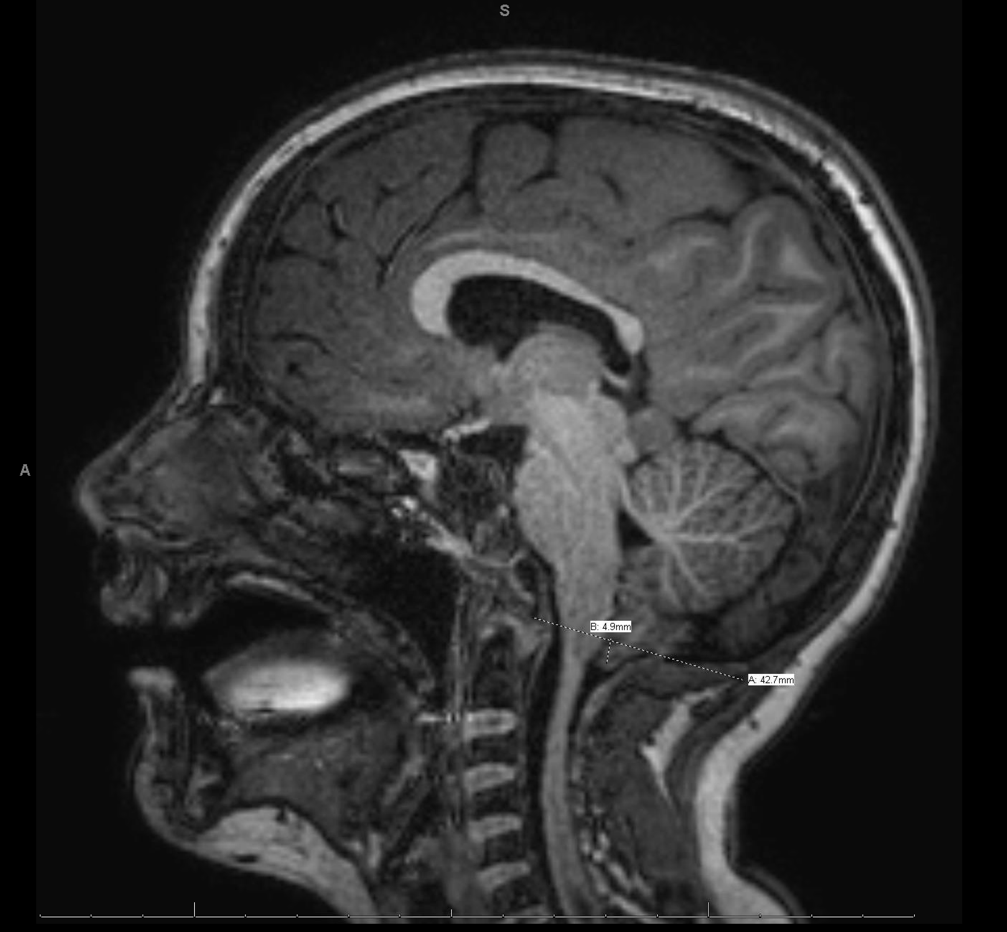
**

**Figure S3. NK-cell cytotoxicity assay with IL-2 stimulation.** Evaluation of NK cell function (Patient vs control sample) with/without IL-2 stimulation. E:T represents ratio of effector NK cells (from PBMCs) to target cells (K562 monocytes lacking MHC class I). % cytotoxicity is measured by ^51^Cr released assay from pre-labeled target cells.


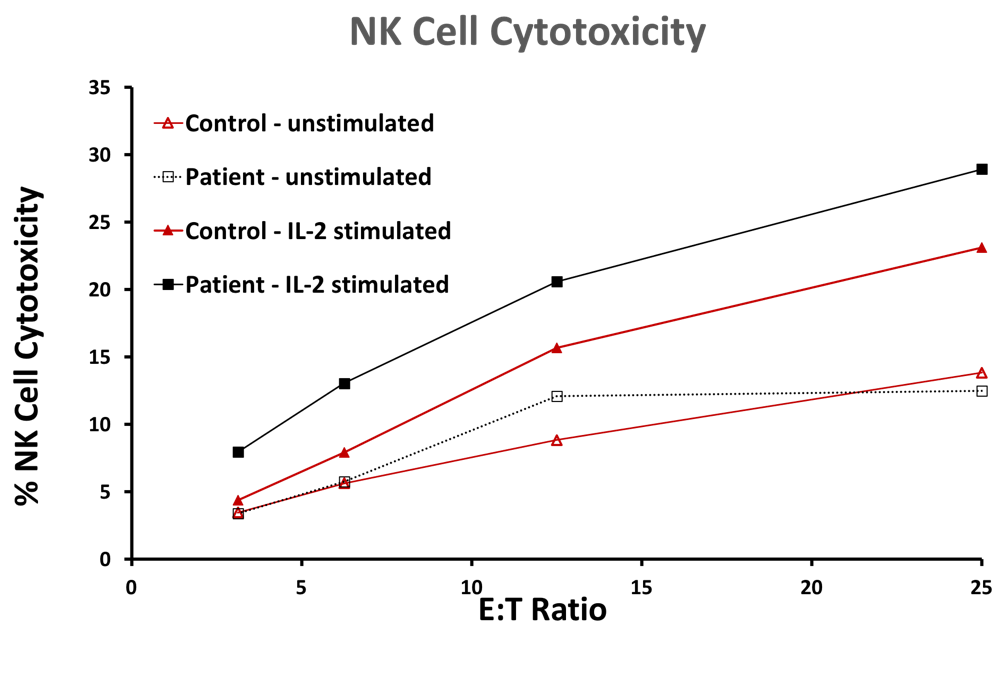

Supplement: Supplementary file 1 [file Data_Sheet_1.docx]
